# Magnitude and pattern of anxiety levels with gender wise predilection of coping strategies amid resident doctors of emergency department

**DOI:** 10.12669/pjms.38.4.5105

**Published:** 2022

**Authors:** Sehrish Zehra, Farhan Muhammad Qureshi, Samira Faiz, Kanwal Khalid

**Affiliations:** 1 Dr. Sehrish Zehra, MP, Department of Community Health Sciences, Karachi Institute of Medical Sciences, Malir Cantt, Karachi, Pakistan; 2 Dr. Farhan Muhammad Qureshi, MS - Public Health & Health Promotion, Department of Community Health Sciences, Karachi Institute of Medical Sciences, Malir Cantt, Karachi, Pakistan; 3 Dr. Samira Faiz, MPH, Department of Community Health Sciences, Karachi Institute of Medical Sciences, Malir Cantt, Karachi, Pakistan; 4 Dr. Kanwal Khalid, MPH, Department of Community Health Sciences, Karachi Institute of Medical Sciences, Malir Cantt, Karachi, Pakistan

**Keywords:** Anxiety, coping strategies, emergency department, GAD-7, resident, doctors, teaching hospitals

## Abstract

**Objectives:**

To screen and assess the severity level of anxiety, its influencing factors along with the gender-wise predilection of coping strategies among resident doctors working in accident and emergency departments.

**Methods:**

A transverse study was conducted amongst 260 resident doctors of accident and emergency department of different teaching hospitals of Karachi from October 2020 until March 2021. A demographic sheet containing questions related to factors, GAD-7 (Generalized Anxiety Disorder) and Brief COPE were used to measure the severity level of anxiety and coping strategies. Data was scored according to the standard scoring procedure for each subscale and for further statistical analysis SPSS Version 21 was used.

**Results:**

Out of all participants, 68.1% were <30 years of age, 63.1% were females while 54.2% were single. The findings of the study showed the prevalence of anxiety within the range of normal (38.1%), mild (35.0%), moderate (16.9%) and severe (10.0%). Gender (p= 0.001), marital status (p= 0.040) and job satisfaction (p= 0.007) among resident doctors were significantly associated with level of anxiety. Deemed to coping strategies, the most frequently were planning (n=145, 90.0%), acceptance (n=141, 87.6%), and religion (n=137, 85.1 %). All coping strategies were mostly opted by females except substance abuse.

**Conclusion:**

More than a half of the resident doctors manifested with mild to severe anxiety disorder, which highlights the need for psychological support programs to minimize anxiety levels and to ensure a healthy environment at workplace for the health practitioners.

## INTRODUCTION

Anxiety is a widespread psychological disorder worldwide, that everyone feels at some point in their life. It may affect mental health and becomes generalized anxiety disorder (GAD) “an excessive and uncontrollable worry”, if happens frequently, resulting in profound personal suffering and financial strain due to inability to accomplish daily tasks.^1^,^2^ Medical field in itself is psychologically demanding^3^ and stressful but dealing with injured and critically ill patients visiting Accident and Emergency Department (A&E) is one of the factors exaggerating emotional stress in health care settings. This stress creates pressure that may lead to anxiety; increasing the risk of lack of concentration and mental exhaustion that ultimately affect clinical performance.^4^

Globally, the main aim of the teaching hospitals is to train resident doctors and produce knowledgeable, skilled and compassionate medical practitioners that are able to treat ailing people through advancement and development in medical field.^5^ In medical profession, anxiety warrants greater attention because of the negative impact on professional development that compromised quality of patient care.^5^ Doctors of A&E department are responsible for the provision of emergency treatment for acutely ill and traumatic patient however, this may be compromised due to scarcity of resources due to large number of patients, constant noise pollution and multi- tasking.^4^

A wide range of researches reported higher prevalence of anxiety with a severity level of moderate to severe in resident doctors of A&E departments.^2^,^6^ Studies conducted in western part of the world demonstrated a range of 3 to 35% of postgraduate medical residents suffering from significant levels of anxiety during their training tenure leading to the extreme act of suicide.^7^ Further, literature exhibited the persistent and progressive stress related anxiety throughout the duration of training or residency.^8^

Coping refers to the opinions, views and actions of an individual to deal with anxiety caused by stressful life events. A variety of coping strategies such as acceptance, self-blame, others blame, planning, positive reframing, use of instrumental support, religious practices, isolation and sleeping etc. were adapted and found useful to reduce anxiety.^9^

To the best of our knowledge, there has not been a local study specifically on anxiety in postgraduate resident doctors of A&E department published in literature. The present study was designed to answer three research questions; (1) What is the prevalence of different levels of anxiety among resident doctors of A&E department? (2) What are the influencing risk factors that cause anxiety in resident doctors of A&E department? and (3) What are the gender-wise distribution of strategies that participants used to cope with anxiety. The selection of residents was determined to identify the problems at an early stage to prevent trainees with progressive levels of anxiety in future. This study bridges the gap by estimating anxiety levels as well as its associated influencing factors in resident doctors of A&E department with coping strategies especially in developing countries in order to provide timely assistance and suitable intervention.

## METHODS

This transverse study was carried out on resident doctors of A&E department in public and private sector teaching hospitals of Karachi, Pakistan from October 2020 until March 2021. Participants were recruited through convenient sampling method. Ethical approval was obtained vide letter Ref #: ERC-KIMS/005/19 from the Ethical Review Committee (ERC) of Karachi Institute of Medical Sciences. Sample size was calculated using Open Epi online sample size calculator. The prevalence of anxiety among resident doctors was taken as 15.9%,^10^ with 95% confidence level and 5% precision, the calculated sample size was 206 that was inflated to 260. Participants were interviewed after an informed consent.

The target population consisted of resident doctors who have an experience of six months or more, irrespective of age and gender. However, participants with history of any diagnosed medical or psychological disorder through self-report were excluded. Similarly, self -reporting pregnant female participants were also excluded. To rule out undiagnosed depression, a depression screening tool PHQ-2 (patient health questionnaire-2)^11^ was used after which we had a morbidity, pregnancy and depression free sample of 260 participants - eligible to participate in the study.

The basic socio-demographic traits like age, gender, marriage status, family system, monthly earnings and other influencing factors of anxiety such as; job satisfaction, peer pressure, sleep time span, regular physical exercise or walk, history of tragic event that might be associated^12^ with anxiety among health care providers were recorded. General Anxiety Disorder-7 (GAD-7) Scale,^13^ a seven-item questionnaire was used to assess the level of anxiety and the “Brief COPE questionnaire”^14^ was used to observe the anxiety related coping strategies. Participants who were found to have anxiety by GAD-7 were asked to fill the Brief COPE Questionnaire to observe the coping strategies they usually opt.

### Statistical Analysis:

All gathered data were entered and analyzed using Microsoft Excel and SPSS version 21. Descriptive analysis was performed of sociodemographic variables as frequencies and percentages. Comparisons for continuous variables were made using t-tests and Analysis of Variance (ANOVA) while for categorical variables, chi-square test was used with the significance level set at 0.05. Anxiety Score taken as categorical outcome was analyzed using logistic regression model.

## RESULTS

A total of 260 resident doctors participated in the study with the mean age of 28.33 ± 2.41 years. Majority of the participants were less than 30 years of age (68.1%), females (63.1%) and doing private practice (25.8%). The descriptive findings of the factors potentially leading to anxiety and their comparison with the level of anxiety taken as no anxiety, mild, moderate and severe are shown in Table-I. Gender (p=0.001), monthly earnings (p=0.026), peer pressure (p<0.001), history of tragic incident (p<0.001), having regular exercise or walk (p<0.001) and job satisfaction (p=0.007) were found to be highly associated with levels of anxiety.

**Table-I:** Potential risk factors with different Level of Anxiety among participants (N=260).

Potential factors of anxiety		Descriptive (N=260)	Anxiety Level (N=260)				

			No (n=99)	Mild (n=91)	Moderate (n=44)	Severe (n=26)	p-value

		n (%)	n (%)	n (%)	n (%)	n (%)	
Age Group (Years)	< 30	177(68.1)	66(37.3)	64(36.2)	31(17.5)	16(9.0)	0.818
	30 or above	83(31.9)	33(39.8)	27(32.5)	13(15.7)	10(12.0)	
Gender	Male	96(36.9)	49(51.0)	34(35.4)	10(10.4)	3(3.1)	0.001
	Female	164(63.1)	50(3.5)	57(34.5)	34(20.7)	23(14.0)	
Marital Status	Single	141(54.2)	49(34.8)	44(31.2)	29(20.6)	19(13.5)	0.040
	Married	119(45.8)	50(42.0)	47(39.5)	15(12.6)	7(5.9)	
Family System	Conjugal	118(45.4)	36(30.5)	39(33.1)	27(22.9)	16(13.6)	0.013
	Joint	142(54.6)	63(44.4)	52(36.6)	17(12.0)	10(7.0)	
Monthly Earnings (Rupees in Thousands)	<50 or >100	129(49.6)	49(38.0)	41(31.8)	19(14.7)	20(15.5)	0.026
	50 - 100	131(50.4)	50(38.2)	50(38.2)	25(19.1)	6(4.6)	
Private Practice	No	193(74.2)	68(35.2)	71(36.8)	37(19.2)	17(8.8)	0.134
	Yes	67(25.8)	31(46.3)	20(29.9)	7(10.4)	9(13.4)	
Sleep Time Span (hours)	< 6 - > 8	109(41.9)	40(36.7)	29(26.6)	23(21.1)	17(15.6)	0.009
	6 - 8	151(58.1)	59(39.1)	62(41.1)	21(13.9)	9(6.0)	
Regular Exercise	No	185(71.2)	56(30.3)	70(37.8)	36(19.5)	23(12.4)	<0.001
	Yes	75(28.8)	43(57.3)	21(28.0)	8(10.7)	3(4.0)	
Job Satisfaction	No	98(37.7)	29(29.0)	37(37.8)	15(15.3)	17(17.3)	0.007
	Yes	162(62.3)	70(43.2)	54(33.3)	29(17.9)	9(5.6)	
Workplace Peer Pressure	No	92(35.8)	52(56.5)	24(26.1)	13(14.1)	3(3.3)	<0.001
	Yes	168(64.2)	47(28.0)	67(39.9)	31(18.5)	23(13.2)	
Tragic Incident History	No	180(69.2)	80(44.4)	66(36.7)	25(13.9)	9(5.0)	<0.001
	Yes	80(30.8)	19(23.0)	25(31.3)	19(23.9)	17(21.3)	

* Includes unmarried, divorced, widowed, separated.

For each potential risk factor, logistic regression was applied to estimate the odds ratio with different levels of anxiety (Table-II). Values present adjusted odds ratios with corresponding 95% confidence intervals. Age group younger than 30 years were observed to have mild anxiety than the older age group (AOR 1.30; 95% CI 0.62, 2.71). On the other hand, being male was found to be protective towards developing anxiety. With the odds of 25.95 (95% CI 3.84, 175.06) and 3.07 (95% CI 1.18, 7.93) single participants were likely to have severe and moderate anxiety respectively. Low household income was also found to be highly associated with severe anxiety (AOR 4.22; 95% CI 0.96,18.55). Participants who were not regular with daily physical exercise had 29 times more chance to develop anxiety than those who exercise regularly. Similarly, lack of job satisfaction was highly associated with severe anxiety (AOR 7.69; 95% CI 1.64, 35.92).

**Table-II:** Logistic Regression Analysis of Anxiety Levels with its Potential Risk Factors (N=260).

Potential Factors of Anxiety	Mild Anxiety		Moderate Anxiety		Severe Anxiety	

	AOR* (95%CI^†^)	p value	AOR* (95%CI^†^)	p value	AOR* (95%CI^†^)	p value
Age Group; <30 years	1.30 (0.62, 2.71)	0.482	0.67 (0.25, 1.81)	0.434	0.06 (0.01, 0.42)	0.005
Gender; Male	0.68 (0.35, 1.32)	0.263	0.36 (0.14, 0.93)	0.035	0.11 (0.01, 0.13)	<0.001
Marital Status; Single	1.07 (0.53, 2.17)	0.832	3.07 (1.18, 7.93)	0.020	25.95 (3.84, 175.06)	0.001
Family System; Conjugal	1.30 (0.65, 2.58)	0.447	2.47 (1.04, 5.85)	0.039	2.69 (0.72, 10.09)	0.141
Monthly Earnings; < 50 & > 100 thousand	0.76 (0.40, 1.45)	0.410	0.59 (0.25, 1.38)	0.227	4.22 (0.96, 18.55)	0.056
Private Practice; No	1.30 (0.62, 2.71)	0.475	2.01 (0.71, 5.64)	0.183	0.12 (0.02, 0.64)	0.016
Sleep Time Span; <6 or >8 hours	0.59 (0.30, 1.17)	0.136	2.00 (0.86, 4.63)	0.106	2.14 (0.57, 7.99)	0.254
Regular Exercise; No	2.31 (1.12, 4.73)	0.022	3.91 (1.14, 10.40)	0.006	29.72 (3.58, 246.17)	0.002
Job Satisfaction; No	1.19 (0.59, 2.41)	0.616	0.67 (0.27, 1.66)	0.392	7.69 (1.65, 35.92)	0.009
Peer Pressure at Work; No	0.35 (0.18, 0.67)	0.002	0.48 (0.20, 1.16)	0.107	0.09 (0.01, 0.56)	0.009
Tragic Incident History; No	0.74 (0.34, 1.59)	0.445	0.34 (0.13, 0.84)	0.021	0.07 (0.01, 0.30)	<0.001

† CI, Confidence intervals,

* AOR, Adjusted odds ratio

The overall prevalence of anxiety level among participant was 35.0% mild, 16.9% moderate while 10.0% had severe anxiety. However, 38.1% of the participants were not having any symptoms of anxiety. Participants scoring high on GAD -7 Scale were asked about different approaches they usually opt to deal with anxiety. The various strategies used to cope with anxiety among participants (n=161) is presented in Table-III. The most frequently being planning (n=145, 90.0%), acceptance (n=141, 87.6%), and religion (n=137, 85.1%). However, the least adapted was substance use (n=12, 7.45%). It was observed that all the coping strategies were mostly opted by female participants except substance abuse.

**Table-III:** Gender-wise Distribution of Coping Strategies used by Study Participants (n=161).

Coping Strategies	n(%)	Males n(%)	Females n(%)	P-Value
Self-distraction	127(78.9)	36(28.3)	91(71.7)	0.648
Active Coping	135(83.9)	32(23.7)	103(76.3)	<0.001
Denial	86(53.4)	18(20.9)	68(79.1)	0.014
Emotional Support	122(75.8)	33(27.0)	89(73.0)	0.290
Positive Reframing	132(82.0)	36(27.3)	96(72.7)	0.253
Planning	145(90.0)	36(24.8)	109(75.2)	<0.001
Humor	100(62.1)	36(36.0)	64(64.0)	0.015
Acceptance	141(87.6)	42(29.8)	99(70.2)	0.659
Religion	137(85.1)	38(27.2)	99(72.3)	0.332
Self-blame	101(62.7)	23(22.8)	78(77.2)	0.020
Venting	106(65.8)	28(26.4)	78(73.6)	0.282
Behavioral Disengagement	104(64.6)	30(28.8)	74(71.2)	0.896
Instrumental Support	119(73.9)	34(28.6)	85(71.4)	0.770
Substance Abuse	12(7.45)	7(58.3)	5(41.7)	0.021

## DISCUSSION

Accident and Emergency department (A&E) is a high pressure and a high intensity unit of any hospital which can be stressful to work leading to increase in the severity of anxiety among healthcare staff especially resident doctors.^15^ Clinical and administrative work of A&E is usually carried out concurrently in order to provide efficient heath care services.^16^ In addition, anxiety secondary to stress is because of severity of patients’ illness, tough working schedule, night shift duties, excessive tiring workloads and high patient flow.^2^,^17^ Moderate to severe level of anxiety has been associated with mental and psychological consequences.^2^ This study found that almost 62% of resident doctors working in A&E department faced varying level of anxiety. Findings of studies conducted in Pakistan and Saudi Arabia are in line with the results of this study and reported most of the healthcare personnel and doctors working in A&E department were suffering from mild to severe levels of anxiety.^2^,^18^ On the contrary, study conducted in Malaysia reported lower prevalence in A&E doctors.^3^

Pertaining to the age groups, those with age less than 30 years showed a higher level of mild anxiety than with age group more 30 years. In support, literature suggested association of young age with generalized anxiety disorders and relate it with the lack of experience and training due to young age,^19^ however, another study found higher levels of anxiety with advanced age group and declared rare in early decades of life.^3^,^20^ Female participants of this research were found associated with higher levels of anxiety in comparison of males. This result is consistent with the findings of other researches and declared females are more prone to develop GAD.^21^,^22^ In contrast, Yahaya et al. reported more anxiety in males than females.^3^ Further, results of this research revealed that job satisfaction is associated with developing different levels of anxiety and has a significant impact on psychological wellbeing.^3^,^23^

In this resident doctors’ cohort, participants mainly using coping strategies such as planning, acceptance, religion, positive reframing, and active coping. However, other published researches reported use of maladaptive strategies i.e., self-distraction, self-blame, denial, substance use and venting.^24^,^25^ On a further note, substance abuse was declared the least common coping strategy that was adapted by resident doctors of A&E department in our research which was consistent with the results of a research conducted in Malaysia that also reported lower rates of substance abuse.^3^

A variety of assessment tools had been used for depression and anxiety related disorders since last few decades. In the past, researchers used screening tools that have lower specificity that might be the reason of reporting high prevalence of GAD and other psychological symptoms in healthcare professionals. Current research revealed high anxiety rates of different levels in resident doctors of A&E department even after use of more reliable and highly specific diagnostic scales such as PHQ-2 and GAD-7, that is the strength of this research.

### Limitations of the study:

The current study only included resident doctors of A&E departments, thus limit the generalizability of the study findings. There is a need to do in depth study on the same issue, preferably cohort study and/or not just focusing on resident doctors of A&E department but other specialties and departments of hospitals.

## CONCLUSION

The prevalence of anxiety was significantly high among resident doctors of A&E department of hospitals in Karachi. This study calls for health promotion practices, strategies and interventions specifically designed for the benefit of all health care workers affiliated with A&E and other hospital departments as well. The early detection and management of GAD will not only reduce the disease burden but will also condense the development of psychological consequences ultimately leading to the betterment of the life of an affected individual.

### Authors’ Contribution:

**SZ:** Conceived, designed and acquisition of data, accountable for the accuracy and integrity of work.

**FMQ:** Study supervisor and drafting of manuscript.

**SF:** Statistical analysis, editing and critical revision of the manuscript for important intellect content.

**KK:** Helped in data collection, analysis and interpretation, final approval of manuscript.
